# Predicting wellbeing over one year using sociodemographic factors, personality, health behaviours, cognition, and life events

**DOI:** 10.1038/s41598-023-32588-3

**Published:** 2023-04-05

**Authors:** Miranda R. Chilver, Elyse Champaigne-Klassen, Peter R. Schofield, Leanne M. Williams, Justine M. Gatt

**Affiliations:** 1 Neuroscience Research Australia, Randwick, NSW 2031 Australia; 2 School of Psychology, University of New South Wales, Sydney, NSW 2052 Australia; 3 School of Medical Sciences, University of New South Wales, Sydney, NSW 2052 Australia; 4 Department of Psychiatry and Behavioral Sciences, Stanford School of Medicine, Stanford University, Stanford, CA 94305-5717 USA; 5 Mental Illness Research Education and Clinical Centers VISN21, Veterans Administration Palo Alto Health Care System, Palo Alto, CA 94304-151-Y USA

**Keywords:** Psychology, Cognitive neuroscience

## Abstract

Various sociodemographic, psychosocial, cognitive, and life event factors are associated with mental wellbeing; however, it remains unclear which measures best explain variance in wellbeing in the context of related variables. This study uses data from 1017 healthy adults from the TWIN-E study of wellbeing to evaluate the sociodemographic, psychosocial, cognitive, and life event predictors of wellbeing using cross-sectional and repeated measures multiple regression models over one year. Sociodemographic (age, sex, education), psychosocial (personality, health behaviours, and lifestyle), emotion and cognitive processing, and life event (recent positive and negative life events) variables were considered. The results showed that while neuroticism, extraversion, conscientiousness, and cognitive reappraisal were the strongest predictors of wellbeing in the cross-sectional model, while extraversion, conscientiousness, exercise, and specific life events (work related and traumatic life events) were the strongest predictors of wellbeing in the repeated measures model. These results were confirmed using tenfold cross-validation procedures. Together, the results indicate that the variables that best explain differences in wellbeing between individuals at baseline can vary from the variables that predict change in wellbeing over time. This suggests that different variables may need to be targeted to improve population-level compared to individual-level wellbeing.

## Introduction

Mental wellbeing refers to a state of optimal functioning that is theorised to consist of two dimensions: subjective (or hedonic) wellbeing which includes feelings of life satisfaction and positive affect balance^1^, and psychological (or eudaimonic) wellbeing which includes a sense of purpose, autonomy, and mastery over one’s environment^2^. Although the two-dimensional approach to wellbeing dominates the literature, their components are highly correlated and past work has found that both subjective and psychological wellbeing load onto a single wellbeing construct^3,4^ and can be measured as such^5^. However, the majority of past research aiming to identify predictors of wellbeing have focused on links with subjective wellbeing only, and many have focused on cross-sectional research designs. Many longitudinal studies focus only on a single domain, such as personality^6^, lifestyle^7,8^, life events^9^, or leisure activities^10^ separately, making it difficult to determine the degree to which these variables explain unique variance in mental wellbeing. Some longitudinal studies have been conducted which do consider multiple domains, but with a primary focus on older adults^11–13^, and the extent to which these results generalise to other stages of adulthood is unclear. The present study aimed to compare the contribution of a wide range of potential wellbeing predictors by utilising data from the TWIN-E study of Wellbeing, a longitudinal twin cohort study^14^. The TWIN-E study was designed to investigate a variety of questions regarding the heritability and neurological basis of mental wellbeing. However, it also provides a rich dataset of sociodemographic, psychosocial, emotional, cognitive, and lifestyle factors which may be used to predict wellbeing on the basis of the biopsychosocial model of mental health^15,16^.

### Associations between psychosocial factors and wellbeing

#### Personality

Personality traits have received significant attention with regards to their relationship to mental wellbeing, and in particular, life satisfaction^6,17–19^, although cross-sectional work has also associated personality traits with psychological wellbeing^20^. Neuroticism and extraversion appear to have the strongest associations with wellbeing^6,19^. Neuroticism may be linked to poorer mental wellbeing because those high in neuroticism tend to have greater difficulty maintaining positive affect balance^19^, are more likely to use maladaptive emotion regulation strategies^21,22^, and more likely to experience negative life events^23^. Meanwhile, extraversion is linked to higher mental wellbeing which may be due its association with more positive affect balance^19^, and a greater tendency to use social support as a coping strategy to reduce stress^24,25^.

The remaining three of the Big Five personality traits, conscientiousness, openness to experience, and agreeableness have also been associated with subjective and psychological wellbeing in both cross-sectional^20^ and longitudinal research^6^. Conscientiousness is likely to be related to wellbeing through its associations with healthy lifestyle and achievement striving^26–28^, and some studies have suggested its link to wellbeing is equally as strong as extraversion^19,20^. Agreeableness and openness tend to have weaker associations with wellbeing, but may contribute to greater personal growth and relationship satisfaction^20,29^. Thus, all of the Big Five personality traits are investigated here.

#### Health and Lifestyle factors

There is growing evidence for a causal link between physical and mental health with meta-analyses indicating that those with better physical health are more likely to maintain better psychological health throughout their life^30,31^. Although this effect has been speculated to be mediated via conscientiousness^27^, longitudinal and intervention studies have found that a healthy diet and regular exercise are associated with improvements in mental wellbeing and reductions in psychological distress^32–35^. Exercise may directly improve mood through increased release of serotonin and beta-endorphins^36^, while diet-related effects are proposed to occur via an increase in complex carbohydrates, B vitamins, and antioxidants^37^. While fruit and vegetable intake appear to be related to better wellbeing^35^, the balance of other food groups is likely also important^32^. Furthermore, as diet and exercise play a key role in weight management, it is also important to consider whether body mass index (BMI) associated with these health-related factors might also explain differences in wellbeing. Some studies have shown, for instance, associations between high BMI and low wellbeing^38^, but these effects have sometimes been attributed to other factors such as poor social support^39^.

Other health-related factors such as sleep, smoking habits, and alcohol consumption may also have impacts on mental wellbeing. Sleep is crucial for maintaining good cognitive function, facilitating the ability to regulate emotions and manage stress^40^. Inadequate sleep can contribute to poor quality of life and mental wellbeing^41^, potentially via other poor health behaviours including higher likelihood of smoking^42^ and higher alcohol consumption^43^. This association may be explained by a shared association with risk-taking behaviours^44^, or by stress decreasing sleep duration and increasing alcohol and tobacco consumption as a coping mechanism^45,46^. Dependence on tobacco has also been independently associated with poorer psychological wellbeing and reduced quality of life^47,48^. In comparison, the association between alcohol consumption and mental wellbeing is less clear and may have inverse short-term and long-term impacts. Drinking alcohol tends to increase positive affect in the moment although it appears to have only minor spill-over effects on general wellbeing^49^, while heavy use is associated with reductions in quality of life, particularly in men^50^. In a healthy non-alcohol dependent population, it is therefore unclear whether alcohol consumption is associated with wellbeing^49^.

#### Work and leisure

Being happy at work is shown to significantly predict subjective wellbeing outside of work^51^, and better psychological wellbeing has been found to predict increases in work performance^52,53^, but whether work performance can predict wellbeing is unclear. However, work performance may be an indicator of higher autonomy at work and the potential for promotions which could improve one’s financial situation and improve wellbeing via increases in income^54^. Thus, the current study also considers the potential predictive relationship of work performance on wellbeing, measured through consideration of absenteeism and the inverse of presenteeism.

Outside of work, leisure time allows individuals to pursue activities that they find intrinsically motivating^55^, that contribute to a sense of purpose or mastery^56^, and provide the opportunity to socialize, and forming meaningful social relationships^57,58^. Spending time with friends and family can be a direct source of happiness and can also provide the social support needed to reduce the impacts of stress^59^. Other activities such as regularly volunteering in the community might also contribute to wellbeing through gaining social support resources, but also by improving one’s sense of community and meaningful contribution^60^. The current study therefore included various measures of leisure activities, including reading, volunteering, and time spent socializing with friends and family.

### Associations between emotion and cognitive processing and wellbeing

Emotion regulation strategies describe the coping methods that people use under stress. Two key strategies include emotional suppression, which describes an attempt to suppress expressions of one’s emotions in response to a stimulus, and cognitive reappraisal, which refers to reappraising events as being less negative or more positive^21^. Both types of emotion regulation strategies are linked to wellbeing, with greater use of emotional suppression being associated with poorer wellbeing compared to greater use of cognitive reappraisal^21,61^. This suggests that cognitive reappraisal could have a direct effect on how life events are interpreted and experienced as either positive or negative, which in turn could impact one’s sense of wellbeing.

Cognitive abilities may impact how easily people are able to apply different emotion regulation strategies^62–64^. Having improved inhibition, cognitive control, cognitive flexibility, and working memory has been associated with an improved ability to regulate one’s emotions both with^63^ and without instruction after receiving negative feedback in laboratory settings^64^. These cognitive functions have also been associated with mental wellbeing when assessed using performance measures like reaction time and accuracy^65,66^. The ability to quickly process and recognize emotional stimuli, especially happy facial expressions, has been associated with higher wellbeing using emotion processing tasks evaluated at the behavioural and neural fMRI level^67,68^. Better facial processing is linked to higher empathy^69^, and may facilitate supportive social interactions which bolster support resources and subsequently wellbeing. Thus, the predictive role of cognitive and emotion processing was also assessed in the current study.

### Recent life events

Apart from lifestyle choices and cognitive factors, the types of recent life events recently experienced, especially major or unexpected events, can also have an impact on wellbeing. Although it may be assumed that experiencing a major negative or positive life event would alter a person’s wellbeing, some early research using cross-sectional data concluded that even events such as winning the lottery or becoming paralysed had relatively small impacts on wellbeing^70^. That is, while significant positive and negative life events can result in initial changes in wellbeing, their effects tend to be limited to a reasonably short timeframe^71^. This adaptation can be explained through the dynamic equilibrium model^72^, whereby an individual’s happiness returns to a set-point level of wellbeing over time. However, a meta-analysis of prospective and post-hoc designs found that certain life events such as having children or becoming unemployed can have lasting impacts on wellbeing^9^. This research also noted significant individual differences in the effects of such events; while some people adapted to their new life circumstances, other people did not. Therefore, how individuals appraise their life events determines the impact of these events^9,73^.

### The current study

The aim of the present study was to pursue a multimodal investigation of the relative contributions of socio-demographics, personality, health and lifestyle behaviours, cognition, and life events to explaining the variance in wellbeing when these contributions are considered in a single model. We considered cross-sectional contributions to wellbeing at a single point in time and predictive contributions to changes in wellbeing over time. Motivated by prior studies of each modality considered separately, we had three main working hypotheses. Firstly, we hypothesised that personality traits would explain the most variance in both the cross-sectional and repeated measures model due to the role of personality in one’s disposition and behaviours^19,27^. In line with previous research, neuroticism, extraversion, and conscientiousness were expected to be the strongest relative predictors, with neuroticism predicting lower wellbeing and extraversion and conscientiousness predicting higher wellbeing^6,20^. Secondly, we hypothesised that lifestyle factors, especially a healthy diet and high exercise frequency, would be the second strongest predictor of wellbeing in the cross-sectional model^32,33^. Thirdly, we hypothesised that life events, especially work, relationship, and traumatic events, would be the second strongest predictor of wellbeing in the repeated measures model, such that events rated more negatively would predict decreases in wellbeing^9^. Within both models, we expected increased leisure time, more use of cognitive reappraisal, better cognitive function, better work performance, and adequate sleep to contribute to higher wellbeing, and we expected use of emotional suppression, increased smoking frequency, and higher absenteeism to predict lower wellbeing.

## Methods

### Participants

Participants were recruited as part of the TWIN-E study with the original aim of assessing heritability of the measured traits, and the sample size was selected to have at least 80% power to detect low heritability^14^. The sample included here includes the 1339 participants from the TWIN-E study who completed both the baseline and the one year follow up components of the study, and the final sample size provides over 80% power to detect small effects of R^2^ = 0.02 for the current analyses. Due to an administrative error, twelve participants were able to access and complete the follow-up questionnaire earlier than scheduled (less than ten months after baseline). These participants were excluded from the analysis leaving 1327 participants (male n = 524). All participants were same-sex monozygotic or dizygotic twins aged between 18 and 61 years (*M* = 40.4, *SD* = 12.6). As part of the TWIN-E study protocol, participants were required to be of European ancestry due to genetic stratification effects, and had English as their primary language^14^. Participants were excluded if they had a current or previous psychiatric illness, a history of stroke or neurological disorder, brain injury, chronic and serious medical conditions, blood-borne illnesses, substance abuse, or uncorrected visual impairments.

Baseline data collection was conducted from 2009 to 2012 and participants were invited to complete the one year follow up from 2010 to 2013. Ethical approval was obtained from the Human Research Ethics Committee of the University of Sydney (03-2009/11430), Flinders University (FCREC#08/09), and the University of New South Wales (HC14256). The research was carried out within the relevant guidelines and in accordance with the Declaration of Helsinki. All participants provided written informed consent prior to enrolment.

### Measures

All data were collected online using a questionnaire battery referred to as WebQ, described previously^5,14^. The primary outcome measure was mental wellbeing, indicated by the COMPAS-W Wellbeing Scale, which was selected because it was developed specifically to assess both hedonic and eudaimonic components of wellbeing as most existing scales of wellbeing measure only one component^5^. The COMPAS-W is a 26-item scale capturing both subjective and psychological components of wellbeing via six subscales of Composure, Own-worth, Mastery, Positivity, Achievement, and Satisfaction. This scale has high internal reliability (0.84) and high test–retest reliability (0.82) in healthy adults^5^, and high internal reliability (0.88) in adolescents aged 12–17 years^74^. The total COMPAS-W score was used as the outcome variable because previous factor analyses of this scale found that all scale items load onto a single factor in addition to the six subscales, thus the total score can provide an overall picture of participants’ composite wellbeing^5^.

The following measures were used as predictors of mental wellbeing. The psychosocial variables examined included: (1) The NEO Five Factor Inventory Revised (NEO-FFI-R) was used to measure Neuroticism, Extraversion, Openness, Conscientiousness, and Agreeableness^75^; (2) Health and lifestyle factors such as diet, sleep, exercise, and social or solo activities were measured using a questionnaire developed for the TWIN-E study, described previously^14,66^ and presented in Supplement 1; and (3) The World Health Organization Health and Work Performance Questionnaire (HPQ) was used to measure absenteeism (time away from work due to illness) and work performance (the inverse of presenteeism) on a scale of 1 to 10 where higher scores indicate higher rates of absenteeism and work performance, respectively^76^. Cognitive and emotional processing was evaluated using (1) the Emotion Regulation Questionnaire to evaluate the emotion regulation strategies of expressional suppression and cognitive reappraisal^21^; and (2) the online WebNeuro test battery included in the TWIN-E study^14,66^ to evaluate performance on tasks evaluating emotional recognition, motor coordination (Finger Tapping test), processing speed (Choice Reaction Time test), inhibition (Go-NoGo test), sustained attention (n-back task), controlled attention (Verbal Interference Test), cognitive flexibility (Attention Switching test), working memory (Digit Span test), recall memory (Recognition test), and executive function (Maze Completion test). Finally, to evaluate recent life events, the Daily Life Events questionnaire developed for the TWIN-E study was used to indicate the occurrence of specific life events in the previous year within the domains of relationships, family, lifestyle, work & finance, and trauma (Gatt et al., 2012). This questionnaire consists of 50 items across all five categories, whereby participants needed to rate the positive or negative impact of each experienced event on a scale from −3 (“Extremely negative impact”) to + 3 (“Extremely positive impact”), with a score of 0 indicating “No impact”. The full scale is provided in Supplement 2.

### Analysis

Cross-sectional and repeated measures multiple regression models were used to assess the relative importance of different wellbeing predictors. In the cross-sectional analysis, two models, Model 1A and Model 1B, were tested initially to assess whether all psychosocial, cognitive and emotion variables contributed significantly to the model. Model 1A included all variables, specifically personality traits (neuroticism, extraversion, openness, conscientiousness, and agreeableness), health-related variables (exercise frequency; dietary intake of fruits and vegetables, red meat, fish, and fast food; nightly sleep duration; BMI; legal substance use including tobacco smoking, alcohol, and caffeine), leisure activity and social factors (marital status, time spent with friends, time spent with family, volunteering frequency, time spent on miscellaneous activities, time spent reading, time spent on mentally challenging tasks such as puzzles, and sporting events), emotion regulation (cognitive reappraisal and emotional suppression), emotion processing (reaction time and accuracy in identifying happy, sad, fearful, angry, disgusted, and neutral facial expressions), cognitive processing (performance on tasks indexing motor coordination, processing speed, inhibition, sustained attention, controlled attention, cognitive flexibility, working memory, recall memory, and executive function), absenteeism and work performance. Age, sex, education, and twin zygosity were included as covariates in the model. The regression model was initially fit as a mixed effects model with family ID included as a random effect to account for twin relatedness, however, as this resulted in singular fit suggesting overfitting of the model, the random effect was dropped and the presented models are multiple regression models.

Model 1A was then compared against Model 1B, a more selective model which only included variables with a strong pre-existing evidence base, including all five personality traits, select health-related variables (exercise frequency, sleep duration, fruit and vegetable intake, BMI, and tobacco smoking), select leisure activities and social factors (marital status, time spent with family, time spent with friends, time spent volunteering, and time spent on miscellaneous activities), both emotion regulation strategies, select emotion and cognitive processing (reaction time for correctly identifying happy facial expressions, motor coordination, inhibition, sustained attention, cognitive flexibility, and working memory), work absenteeism and work performance, and all four covariates (age, sex, education, and twin zygosity).

Model 1A and Model 1B were compared using a tenfold cross-validation procedure with 200 replications using the *xvalglms* package in *R*^77^. The average root mean squared error (RMSE) across cross-validation replications was used to compare the models whereby a lower RMSE indicated better model fit. Once the best-fitting model was identified, the contribution of each predictor to the model was assessed by estimating the delta r^2^ by dropping each predictor individually from the selected model and observing the change in R^2^. Delta r^2^ values indicate the degree to which each predictor contributes unique variance to the model. To estimate how much variance each predictor would account for on its own, delta r^2^ was also calculated relative to a covariates-only model in which only the variable being tested and the covariates (age, sex, education, and zygosity) were included in the reference model.

A repeated measures analysis was also conducted using a similar process. Again, two models were compared, Model 2A and Model 2B, which included all of the same predictors as Model 1A and Model 2B, respectively, but follow-up wellbeing (one year later) was used as the criterion and baseline wellbeing was added as a covariate in both models. In addition, Models 2A and 2B also included recent life events, which were positive or negative events that occurred between baseline and follow-up in the domains of relationships, family, lifestyle, work & finance, and trauma. The models were again compared using a ten-fold cross-validation procedure with 200 replications, and the model with the lower RMSE was the selected model. As with the cross-sectional models, the change in R^2^ was measured relative to the final selected model and a covariate-only model (including age, sex, education, zygosity and baseline wellbeing) to indicate the contribution of each variable in predicting wellbeing.

## Results

### Sample characteristics

Of the 1327 total participants, 208 were unemployed and did not have measures available for the HPQ, and a further 96 participants (8%) had missing data for one or more cognition variables (5% to 14% of cognitive variables missing). These participants were excluded from the analysis, reducing the total sample to 1023 participants. Finally, due to the low number of widowed participants (*n* = 6), these participants were also excluded, resulting in a final sample of 1017 participants. Of these, 420 (41.3%) were male. Most participants (*n* = 705, 69.3%) reported that they had completed at least a graduate degree or diploma, and more than half of the participants were married at baseline (*n* = 536, 52.7%). The average time between baseline and follow up was 15.7 months (*SD* = 5.07). The mean wellbeing score was similar at baseline (*M* = 100.4, *SD* = 10.6) and at follow up (*M* = 100.4, *SD* = 11.1). The overall mean change in wellbeing was minimal (*M* = −0.02) but there was significant variation in the change in wellbeing between participants (*SD* = 6.79).

Further sample characteristics, including means and distributions for each predictor are provided in Tables S1 and S2. Scale reliabilities are provided in Table S3.

### Cross-sectional model results

#### Demographic associations with wellbeing

**Figure 1:**
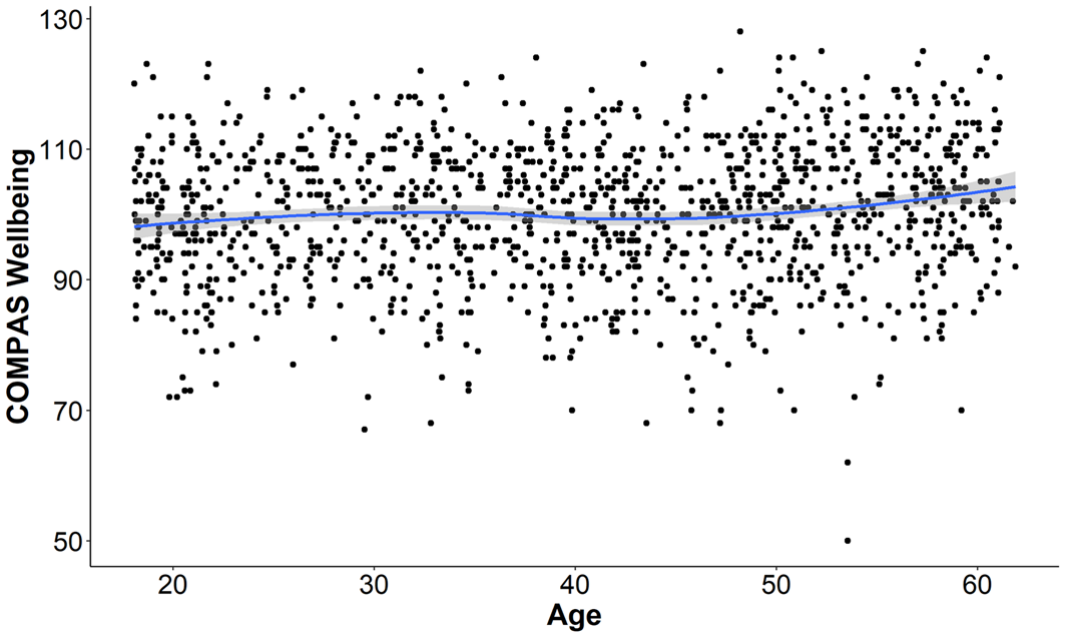
Scatterplot and loess line showing relationship between age and mental wellbeing. The trend line shows a slight cubic increase in wellbeing, with an initial increase to mid-thirties followed by a dip between 40 and 50 years, followed by a final increase to age 65.

**Figure 2:**
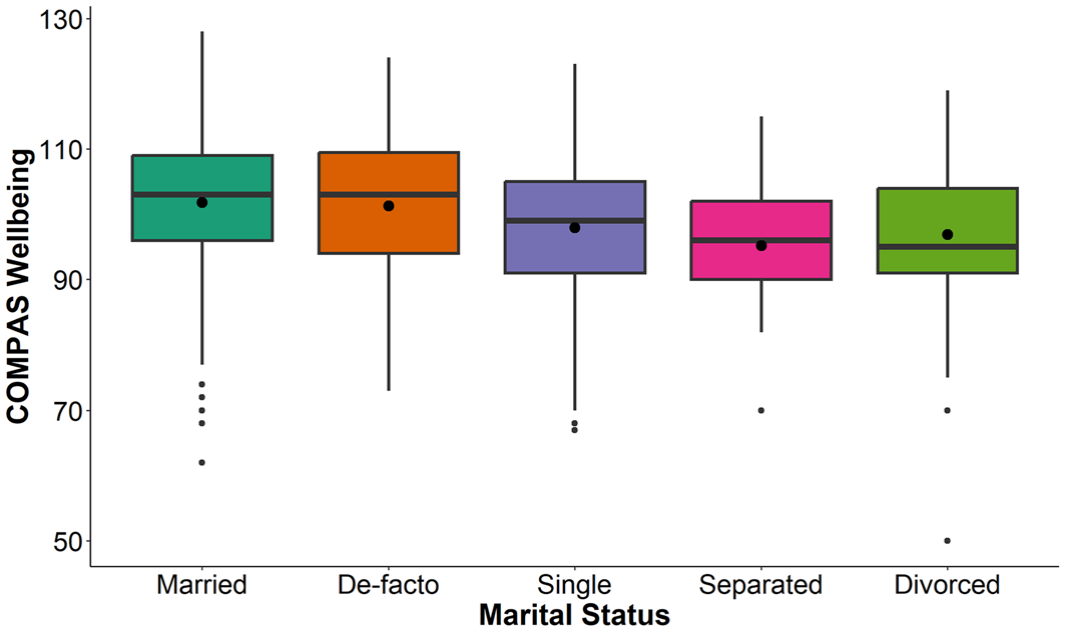
Boxplot showing relationship between marital status and mental wellbeing. Mean wellbeing is indicated by circles inside each boxplot. Pairwise comparisons found that de-facto individuals had higher average wellbeing than singles, and that married individuals had higher average wellbeing than singles, divorcees, and separated individuals.

An initial set of simple regression models and t-tests were used to test for how age, sex, and marital status were related to wellbeing. There was no significant effect of sex (*t* = 0.91, *p* = 0.364), but there was a significant association between wellbeing and age, showing significant linear (*b* = 0.08, *t* = 3.50, *p* < 0.001), quadratic (*b* = 0.001, *t* = 3.70, *p* < 0.001), and cubic (*b* < 0.001, *t* = 3.87, *p* < 0.001) trends shown in Fig. 1. When linear, quadratic, and cubic trends were tested in the same model, the cubic trend was significant (*b* = 0.0003, *t* = 1.98. *p* = 0.047), while the linear and quadratic trends were marginal (*p* < 0.10). There was a significant overall test for marital status (*F* = 10.55, *p* < 0.001). Pairwise comparisons for marital status showed that married individuals had higher wellbeing on average than divorced, separated, or single individuals, and de-facto partners had higher wellbeing than singles. Marital status effects are shown in Fig. 2.

#### Model selection

The tenfold cross-validation procedure comparing Model 1A and Model 1B showed better fit for Model 1B with a lower mean RMSE of 5.17 across 200 replications relative to Model 1A which had a mean RMSE of 5.31. The multiple R^2^ for Model 1B was 0.79 indicating that the model explained 79% of the observed variance in wellbeing, and was similar to Model 1A which had a multiple R^2^ of 0.80. The variance inflation factors were inspected and all were below 2. The relative importance of each predictor in the model was assessed using delta r^2^ (summarised in Table 1).

**Table 1:** Delta R^2^ for predictors of baseline wellbeing (cross-sectional model) and follow-up wellbeing (repeated measures model) relative to the selected and covariate-only models.

Predictor	Cross-sectional model		Repeated measures model	
	Δ R2	Δ R2	Δ R2	Δ R2
	vs. Model 1B	vs. covariates	vs. Model 1B	vs. covariates
Baseline wellbeing^+^			**0.0712**	**0.6153**
Age*	0.0014	0.0054	0.0010	0.0017
Sex*	0.0002	0.0002	0.0007	0.0000
Education*	0.0011	0.0342	0.0006	0.0006
Zygosity*	0.0005	0.0011	0.0005	0.0003
Neuroticism	**0.1102**	**0.5357**	0.0009	0.0015
Extraversion	**0.0423**	**0.3887**	**0.0061**	**0.0082**
Conscientiousness	**0.0557**	**0.3184**	**0.0023**	**0.0024**
Agreeableness	0.0001	0.1462	0.0000	0.0004
Openness	0.0045	0.0289	0.0009	0.0008
Exercise days per week	0.0003	0.0312	**0.0026**	**0.0038**
Fruit and vegetable intake	0.0004	0.0197	**0.0029**	**0.0040**
Body mass index	0.0008	0.0312	0.0011	0.0014
Sleep hours per night	0.0002	0.0125	0.0019	0.0040
Smoking status	0.0014	0.0106	0.0007	0.0008
Leisure time	0.0015	0.0072	**0.0019**	**0.0028**
Friend visits	0.0006	0.0268	0.0018	0.0030
Family visits	0.0003	0.0015	0.0005	0.0004
Marital status	0.0014	0.0228	0.0009	0.0017
Volunteering	0.0007	0.0072	0.0006	0.0005
Absenteeism	0.0000	0.0017	0.0000	0.0000
Work performance	0.0003	0.0556	0.0016	0.0033
Cognitive reappraisal	**0.0160**	**0.1522**	0.0007	0.0007
Emotional suppression	0.0006	0.0788	0.0000	0.0008
Happy reaction time	0.0000	0.0053	0.0000	0.0000
Motor coordination	0.0003	0.0091	0.0002	0.0006
Inhibition	0.0000	0.0063	0.0002	0.0000
Sustained attention	0.0000	0.0014	0.0009	0.0004
Cognitive flexibility	0.0010	0.0135	0.0010	0.0016
Working memory	0.0017	0.0114	0.0004	0.0000
DLE–Relationships^			0.0016	0.0039
DLE–Family^			0.0001	0.0022
DLE–Work^			**0.0043**	**0.0097**
DLE–Lifestyle^			0.0007	0.0060
DLE–Trauma^			**0.0040**	**0.0068**

*Variables included as covariates. ^+^Included as covariate in follow-up model only. ^^^Daily Life Events refer to events or changes occurring between Time 1 and Time 2 and are thus only included in the repeated measures model. Variables predicting at least at least 0.2% of unique variance in each model are indicated in bold. N = 1017.

**Figure 3:**
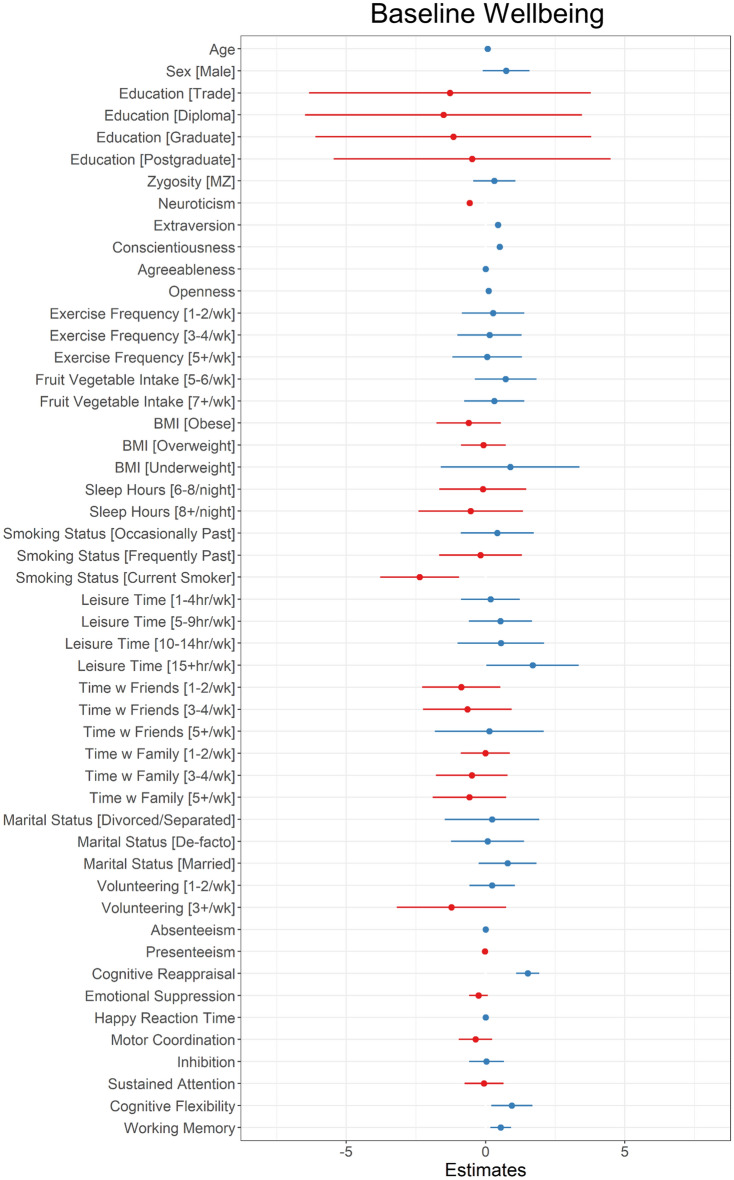
Unstandardised coefficients and 95% confidence interval limits for final cross-sectional model. Blue lines indicate positive coefficients and red lines indicate negative coefficients. Education reference level is High School education; Zygosity reference level is DZ, Marital Status reference level is Single, BMI reference level is Healthy, all other ordinal variable reference levels are “Never” or the lowest level measured. N = 1017, n for ordinal variables provided in Table S2.

In the final cross-sectional model (Model 1B), three personality traits had the greatest predictive value relative to other predictors with delta r^2^ values of 0.11 for neuroticism, 0.06 for conscientiousness, and 0.04 for extraversion in the context of the selected model, and 0.54 for neuroticism, 0.32 for conscientiousness, and 0.39 for extraversion in the covariates-only model. Neuroticism was negatively associated with mental wellbeing, while extraversion and conscientiousness were positively associated. The next strongest predictor was cognitive reappraisal, which had a delta r^2^ of 0.02 in the context of the selected model and 0.15 in the covariate only model. Greater use of cognitive reappraisal was associated with higher wellbeing. The remaining variables each predicted less than 0.01 of unique variance in wellbeing in the selected model. Coefficients and 95% confidence intervals are shown in Fig. 3. Due to the large number of variables in the model, the coefficients should be interpreted with caution.

### Repeated measures model results

#### Model selection

**Figure 4:**
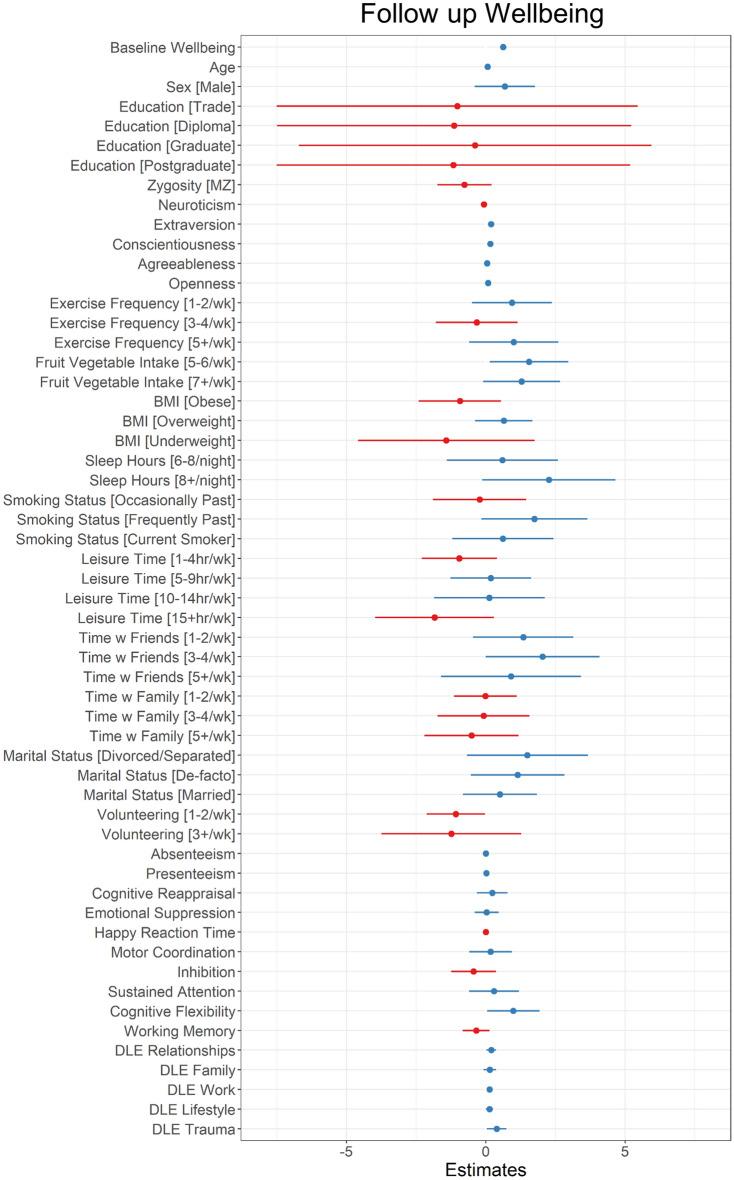
Unstandardised coefficients and 95% confidence interval limits for the final repeated measures model. Blue lines indicate positive coefficients and red lines indicate negative coefficients. Education reference level is High School education; Zygosity reference level is DZ, Marital Status reference level is Single, BMI reference level is Healthy, all other ordinal variable reference levels are “Never” or the lowest level measured. N = 1017, n for ordinal variables provided in Table S2.

In the repeated measures analysis, the ten-fold cross-validation procedure also showed that Model 2B had better fit with a lower mean RMSE of 6.48 across replications compared to Model 2A, which had a mean RMSE of 6.66. The multiple R^2^ for Model 2B was 0.70 in the full sample, indicating 70% of the variance explained in wellbeing change over one year. Again, this was similar to Model 2A which had a multiple R^2^ of 0.71. All variance inflation factors were below 2 with exception to age (VIF = 2.08) and baseline wellbeing (VIF = 4.75). To identify the key predictors, delta r^2^ was assessed for each predictor, provided in Table 1. Baseline wellbeing accounted for the greatest amount of variance with delta r^2^ of 0.071 in the context of the final model, and 0.615 in the covariate-only model. Among the predictors, extraversion explained the most variance in wellbeing change over one year with a delta r^2^ of 0.006 in the context of the selected model and 0.008 in the covariate-only model. Daily life events made the next largest contribution to the model, with changes to work and income having a delta r^2^ = 0.004 relative to the selected model and delta r^2^ = 0.010 relative to covariates only, and traumatic events explaining a similar amount of variance with delta r^2^ = 0.004 in the selected model and delta r^2^ = 0.007 in the covariate-only model. These effects were positively associated with wellbeing based on the rating of how positive or negative each event was; that is, more negative traumatic or work experiences predicted larger decreases in wellbeing, whereas more positively rated events had a positive impact on wellbeing. Other predictors explaining at least 0.2% of unique variance in wellbeing included exercise frequency, conscientiousness, leisure activities, and fruit & vegetable consumption (see Table 1 for details). Each of these factors showed a positive association with wellbeing, as shown in Fig. 4.

## Discussion

A growing body of research demonstrates that mental wellbeing is associated with sociodemographic, psychosocial factors, emotional and cognitive processing and recent life events. The aim of this study was to clarify the relative predictive value of each variable in explaining differences in wellbeing between individuals and change in wellbeing over one year. The results indicate that while personality traits (i.e., neuroticism, extraversion, and conscientiousness) and emotion regulation strategies (especially cognitive reappraisal) were stand out predictors of an individual’s wellbeing relative to other individuals, they were not necessarily the same predictors of an individual’s wellbeing change over time. Extraversion was the strongest predictor of change in wellbeing over one-year period, followed by the extent to which the individual perceived themselves to have experienced either highly positive or negative life events in the domains of work and trauma in the past year. Following these, baseline exercise frequency and conscientiousness predicted changes in wellbeing. Thus, the current study indicates that while personality traits are useful predictors of wellbeing, recent life events and health behaviours are also important. Notably, different sets of predictors appear to explain differences in wellbeing between individuals at baseline compared to those that explain change in wellbeing over time.

There is a large evidence base for the role of personality traits in explaining mental wellbeing, with numerous studies indicating that high neuroticism and low extraversion are risk factors for poor wellbeing and mental illness^17,78^. Neuroticism shares some components with mental wellbeing and mood disorders in that it is characterised by emotional instability and poor emotion regulation^19,79^. Higher neuroticism is also associated with the experience of early life trauma and objective negative events in adolescence and adulthood^23,79^. Previous research has indicated that stressors in critical developmental periods including childhood and adolescence contribute to the emergence of neuroticism, and interventions during these critical periods could reduce the burden of mental illness and poor wellbeing^79^. Parent-training and other psychological interventions have shown promise in reducing neuroticism in past research^80^. Such interventions could be used to clarify whether personality traits play a causal role in wellbeing.

Both extraversion and conscientiousness were also significant predictors of both between-person differences in wellbeing and change in wellbeing over time. Extraversion was the strongest unique predictor of change in wellbeing over one year while covarying for all other predictors. Extraversion has been linked to perceived social support, which has in turn been shown to reduce perceived stress^24^. Longitudinal studies examining the role of personality in transitional periods, such as retirement, have shown that having higher extraversion can help protect against stress and negative impacts on wellbeing that often occur during such periods^18^. Thus, the tendency for individuals higher in extraversion to have more abundant social connections may facilitate maintenance or improvement in wellbeing over time. Similarly, conscientious individuals tend to be more health conscious^27,81^ and goal directed in their work and personal life^26,82^. Over time, this may help contribute to better long-term health, avoiding potential negative effects on mental wellbeing, and better financial security and goal achievement over time. Future research should clarify the relative roles of extraversion and conscientiousness in maintaining and improving mental wellbeing to help direct future interventions.

There has been some debate over the role of life events and lifestyle in mental wellbeing. It is likely that the impact of life events on wellbeing is contextual, such that when an experience is much more positive or negative than typical events, there will be a change in wellbeing, but the degree to which different events are typical can also change over time^72^. The effect of life events on wellbeing also appears to be short-lived. In a two-year longitudinal study, Suh et al.^71^ found that only events occurring in the past three months meaningfully impacted one’s current wellbeing. In the current study, only events from the past 12 months were considered, and no distinction was made according to the amount of time passed since each event occurred, however, there was a focus on the subjective experience of each event as being positive or negative. Based on these subjective ratings, traumatic and work-related events were strong predictors of change in wellbeing over time when accounting for all other variables in the model. Allowing participants to provide their own ratings of experienced life events means that the events with the strongest ratings drive the effect size, and the same objective event can be considered as either positive or negative according to the individual. For instance, events generally perceived as positive (e.g., marriage) could be rated as having a subjectively negative impact, while events generally perceived as negative (e.g., divorce) could be rated as subjectively positive. As such, this method forgoes assumptions about the valence of different life events, while also considering the intensity of each event on an individual level. Thus, the current results show that how an individual appraises recent life events is a good predictor of their future wellbeing.

Interestingly, using cognitive reappraisal as an emotion regulation strategy was another important predictor of between-individual variance in wellbeing, but was not a strong predictor of change in wellbeing over time. Previous research has indicated that using cognitive reappraisal can help reduce the experience of negative emotions by appraising a given situation as less stressful than it might otherwise appear^21^. Because the current research included appraisals of recent life events as predictors of wellbeing, this measure might have captured aspects of cognitive reappraisal as individuals who more readily use that strategy would be expected to have rated their experience of life events more positively. The appraisals of recent life events might similarly have captured aspects of neuroticism related to the experience of more negative life events, reducing the unique predictive strength of neuroticism in the longitudinal model. Future research could help elucidate the relationships between the experience and appraisal of life events, personality traits, and emotional regulation strategies.

Lifestyle factors including sleep, exercise, and diet-related variables explained little variance at baseline but explained more variance one year later, with exercise and fruit and vegetable consumption being the strongest lifestyle predictor. This is consistent with previous cohort studies that associated fruit and vegetable intake with longitudinal increases in mental wellbeing^33,35^. Eating a balanced diet may contribute to wellbeing through obtaining mood-related micronutrients^37^, and might also be a general indicator of health behaviour which would, similar to exercise, reduce the likelihood of chronic illness over time. Although conscientiousness has been proposed to explain the link between wellbeing and health behaviours^27^, the current models accounted for both conscientiousness and lifestyle factors and found that both significantly contributed to wellbeing outcomes. Experimental research manipulating health related behaviours while controlling for personality can shed further light on the link between health and wellbeing, though to date there are promising indications of a causal relationship between physical activity and diet and improvements in wellbeing^32,83^.

The current study made use of the rich dataset provided by the TWIN-E study^14^ to investigate the predictive value that psychosocial factors, emotional and cognitive processing, and recent life events have in terms of predicting wellbeing. However, some limitations are worth noting. Due to the number of variables tested, the coefficients for each and their relative effect sizes may be impacted by many small to moderate correlations with other variables. However, the variance inflation factors were all kept below five (see Table S5 for correlations). As such, delta r^2^ was used to quantify and compare the importance of each predictor to the models. Further research should aim to clarify whether the strongest predictors identified here are simply associative or causal in their impacts on wellbeing. Another limitation is that we only measured life events that occurred from baseline to follow-up, but not prior to baseline. This means we could not evaluate how life events explain differences in wellbeing at baseline, although prior research has indicated that life events are relatively poor at explaining between-individual differences in wellbeing^71^. Future work should nonetheless test these comparisons directly. Finally, the present study uses only two waves of data. Longitudinal studies with three or more waves of data are more informative as they can more clearly separate measurement error from true change, and can identify non-linear trends that cannot be observed in only two waves^84^.

This study examined the unique contributions of psychosocial, cognitive, and life event factors in explaining differences in wellbeing between individuals, and when examining changes in wellbeing over time. Neuroticism, extraversion, conscientiousness, and the use of cognitive reappraisal were the most significant predictors of wellbeing in the cross-sectional model. In comparison, extraversion, the appraisal of work-related and traumatic life events over the past year, exercise frequency, conscientiousness, and fruit and vegetable intake were the strongest predictors of *change* in wellbeing over time after accounting for baseline wellbeing. These findings suggest that the variables associated with differences in wellbeing between individuals, versus those predicting change in wellbeing over time are not always the same, and could therefore be differentially targeted in future health interventions.

## Supplementary Information

Supplementary Information 1.

Supplementary Information 2.

## Data Availability

The data and code used during the current study are available from the corresponding author (JMG) upon reasonable request.
