# Supplementary material for: Predicting wellbeing over one year using sociodemographic factors, personality, health behaviours, cognition, and life events
Source: Sci Rep. 2023 Apr 5;13:5565. doi: 10.1038/s41598-023-32588-3 (PMC10076502; doi:10.1038/s41598-023-32588-3)
Supplement: Supplementary file 1 — Supplementary Information 1. [file 41598_2023_32588_MOESM1_ESM.docx]

**Supplementary Materials**

for

**Predicting wellbeing over one year using sociodemographic factors, personality, health behaviours, cognition, and life events**

Miranda R. Chilver^1,2^, Elyse Champaigne-Klassen^1^, Peter R. Schofield^1,3^, Leanne M. Williams^4,5^^, Justine M. Gatt^1,2^*^.

^1^ Neuroscience Research Australia, Randwick, New South Wales, 2031, Australia

^2^ School of Psychology, University of New South Wales, Sydney, New South Wales, 2052, Australia

^3^ School of Medical Sciences, University of New South Wales, Sydney, New South Wales 2052, Australia

^4^ Department of Psychiatry and Behavioral Sciences, Stanford School of Medicine, Stanford University, Stanford, California 94305-5717, United States of America

^5^ Mental Illness Research Education and Clinical Centers VISN21, Veterans Administration Palo Alto Health Care System, California, 94304-151-Y, United States of America

^Shared senior authors

***Corresponding author:** Associate Professor Justine Gatt; Phone (+612 93991812); Postal address: Neuroscience Research Australia, Barker St, Randwick Sydney NSW 2031 Australia. E-mail address: [j.gatt@neura.edu.au](mailto:m.chilver@neura.edu.au)

**Word count:**

Abstract: 200

Manuscript: 5233

Tables: 1

Figures: 4

Supplementary Materials: 2

References: 75

**Contents:**

**Supplement 1: Health and Lifestyle Questionnaire**

**Supplement 2: Daily Life Events (DLE) Questionnaire**

**Table S1: Sample characteristics for continuous variables**

**Table S2: Sample characteristics for categorical variables**

**Table S3: Reliabilities for multi-item measures**

**Table S4: Final cross-sectional model results**

**Table S5: Final repeated measures model results**

**Table S6: Pearson *r* correlations among all study variables (Excel file)**

**Table S7: Pearson correlation p-values among all study variables (Excel file)**

**Supplement 1: Health and Lifestyle Questionnaire**

| **Exercise** | On average, how many times a week would you exercise (e.g. strength training, aerobic exercise, or activities such as running, swimming, strenuous walking or bike riding) for at least 30 minutes a day? | None  1-2 times  3-4 times  5-6 times  Daily |
| --- | --- | --- |
| **Fruit & vegetable intake** | On average, how many times a week would you eat fresh fruit or vegetables? | None  1-2 times  3-4 times  5-6 times  Daily |
| **Red meat intake** | On average, how many times a week would you eat red meat? | None  1-2 times  3-4 times  5-6 times  Daily |
| **Fish intake** | On average, how many times a week would you eat fish? | None  1-2 times  3-4 times  5-6 times  Daily |
| **Fast food intake** | On average, how many times a week would you eat fast foods? | None  1-2 times  3-4 times  5-6 times  Daily |
| **Sleep per night** | How many hours of sleep do you usually get per night? | Less than 3 hours  3-5 hours  6-8 hours  Over 8 hours |
| **Smoking status** | How frequently do you smoke (cigarettes, tobacco, cigars, pipe)? | Never  Not currently but occasionally in the past  Not currently but frequently (daily) in the past  10 or less per week  11-20 times a day  21-30 times a day |
| **Alcohol consumption** | How frequently do you consume alcohol? | Never  Not currently but occasionally in the past  Not currently but frequently in the past  Once a month or less  2-4 times a month  2-3 times a week  4 or more a week |
| **Caffeine consumption** | On average, how many cups of caffeinated drinks (coffee, coke or energy drinks) would you drink per day? | None  1-2 cups  3-4 cups  Over 5 cups |
| **Leisure time** | On average, how much time in a week would you spend on leisure activities outside of work – e.g., taking community/evening classes, practicing a skill, getting lessons (e.g., dance, music, a new language)? | None  0-1 hour  2-4 hours  5-9 hours  10-14 hours  15 hours or more |
| **Reading time** | On average, how much time in a week would you spend reading (e.g. a book, magazine, other article)? | None  0-1 hour  2-4 hours  5-9 hours  10-14 hours  15 hours or more |
| **Challenges/puzzles** | On average, how much time in a week would you spend on mentally challenging games or puzzles (e.g. chess, crossword puzzles, sudoku, cards or other board games)? | None  0-1 hour  2-4 hours  5-9 hours  10-14 hours  15 hours or more |
| **Time with friends** | On average, how many times a week would you socialise with friends? | None  1-2 times  3-4 times  5-6 times  Daily |
| **Time with family** | On average, how many times a week would you visit your family? | None  1-2 times  3-4 times  5-6 times  Daily |
| **Volunteering** | On average, how many times a week would you volunteer or participate in a community activity? | None  1-2 times  3-4 times  5-6 times  Daily |

***Note.*** To reduce impact of unequal group size, response options for some categories were collapsed into categories shown in Table S2.

**Supplement 3: Daily Life Events questionnaire**

**Instructions**

Below is a list of events which may bring about changes in the lives of those who experience them. Please only consider events that have occurred **in the past 12 months (since your last test session with us)**.

For every event, please indicate

(1) Whether the event did or did not occur,

(2) If the event occurred, rate the extent to which you view the event as having either a positive or negative impact on your life at the time the event occurred on the following scale:

| -3 | -2 | -1 | 0 | 1 | 2 | 3 |
| --- | --- | --- | --- | --- | --- | --- |
| Extremely negative impact | Moderately negative impact | Somewhat negative impact | No impact | Somewhat positive impact | Moderately positive impact | Extremely positive impact |

(3) WHEN the event occurred (how many months ago did this event last occur?)

**Interpersonal relationships**

1. Engagement
2. Marriage
3. Pregnancy (you or your spouse)
4. Pregnancy difficulties (e.g., miscarriage, abortion, delays in conceiving)
5. Sexual difficulties
6. Spouse beginning or ceasing work (loss of job, new job, retirement)
7. Death of spouse
8. Divorce
9. Marital separation
10. Temporary separation from spouse due to work, travel, etc.
11. Major increase in the number of arguments with spouse
12. Marital reconciliation (making up with partner)

**Family life**

1. Death of a close family member or close friend
2. Major change in health or behaviour of family member
3. Gaining a new family member (e.g., through birth, adoption or marriage)
4. Leaving home for the first time
5. Son or daughter leaving home (e.g., due to marriage, university, moving out)
6. Trouble with the in-laws
7. Major change in the number of family get-togethers

**Work, education and finance**

1. Change to a different line of work
2. Being fired, terminated or made redundant
3. Retirement from work
4. Major business readjustments
5. Major change in responsibilities at work (promotion, demotion, transfer, etc.)
6. Troubles with supervisor, boss or superiors
7. Major change in working hours or conditions
8. Major change in financial state (much better or worse off)
9. Taking on a mortgage or loan
10. Foreclosure on a mortgage or loan
11. Purchase of a car or other big purchase
12. Outstanding personal achievement
13. Begin or end of schooling
14. Change to a new secondary or tertiary school
15. Minor violations of the law (e.g., traffic offenses, parking tickets)

**Home, lifestyle and social activities**

1. Major change in living conditions (e.g., building, renovating, or deterioration of home)
2. Change of residence
3. Revision of personal habits (e.g., smoking, drinking, etc.)
4. Major change in type or amount of recreation/sport
5. Major change in church activities
6. Major change in social activities (e.g., attending parties, movies, visiting friends)
7. Major change in sleeping habits
8. Major change in eating habits
9. Took a vacation or holiday

**Traumatic life events**

1. Detention in jail or other institution
2. Involved in direct combat experience in war
3. Involved in a fire, flood, or other natural disaster
4. Witnessed someone else being seriously injured or attacked
5. I was seriously injured, attacked, or assaulted
6. Major personal injury or illness
7. Experienced a stressful or upsetting event that is not otherwise indicated here

**Table S1.** Sample characteristics for continuous variables.

| **Variable** | **M (SD)** |
| --- | --- |
| Time 1 Wellbeing | 100.33 (10.6) |
| Time 2 Wellbeing | 100.35 (11.1) |
| Age | 39.58 (12.2) |
| Neuroticism | 16.18 (7.9) |
| Extraversion | 29.46 (6.3) |
| Openness | 27.59 (6.3) |
| Agreeableness | 34.55 (5.2) |
| Conscientiousness | 34.89 (5.7) |
| Absenteeism (absolute) | 2.61 (43.5) |
| Presenteeism (absolute) | 79.32 (15.4) |
| Cognitive Reappraisal | 5.08 (0.9) |
| Emotional Suppression | 3.49 (1.2) |
| Motor Coordination | 0.25 (0.6) |
| Processing Speed | 0.24 (0.8) |
| Inhibition | 0.13 (0.6) |
| Sustained Attention | -0.07 (0.6) |
| Controlled Attention | -0.12 (0.7) |
| Cognitive flexibility | 0.11 (0.5) |
| Working Memory | -0.35 (1.0) |
| Recall Memory | 0.23 (0.7) |
| Executive Function | 0.28 (0.8) |
| Happy Accuracy | 98.9 (4.2) |
| Fear Accuracy | 81.67 (18.4) |
| Angry Accuracy | 58.6 (16.5) |
| Disgust Accuracy | 47.78 (17.2) |
| Sad Accuracy | 71.1 (21.8) |
| Neutral Accuracy | 87.6 (13.2) |
| Happy RT | 1524.3 (367.2) |
| Fear RT | 3335.21 (1185.3) |
| Angry RT | 2996.54 (1001.6) |
| Disgust RT | 2914.08 (922.8) |
| Sad RT | 2809.24 (1031.0) |
| Neutral RT | 1881.83 (676.6) |

**Table S2.** Sample characteristics for categorical variables

| **Characteristic** | | **n (%)** |
| --- | --- | --- |
| Sex | Female | 601 (59%) |
|  | Male | 422 (41%) |
| Education | Secondary/High school | 239 (23%) |
|  | Trade qualification | 73 (7%) |
|  | Diploma/Certificate | 217 (21%) |
|  | Graduate (degree) | 296 (29%) |
|  | Postgraduate (higher degree) | 194 (19%) |
|  | Other | 4 (0.4%) |
| Zygosity | Dizygotic | 361 (35%) |
|  | Monozygotic | 656 (64%) |
| Exercise Frequency | None | 177 (17%) |
|  | 1-2 per week | 316 (31%) |
|  | 3-4 per week | 306 (30%) |
|  | 5+ per week | 224 (22%) |
| Fruit & Vegetable intake | 4 or less | 189 (18%) |
|  | 5-6 per week | 317 (31%) |
|  | 7+ per week | 517 (51%) |
| Red meat intake | Less than 2 per week | 410 (40%) |
|  | 3-4 per week | 477 (47%) |
|  | 5+ per week | 136 (13%) |
| Fish intake | None | 222 (22%) |
|  | 1-2 per week | 700 (68%) |
|  | 3+ per week | 101 (9.9%) |
| Fast food intake | None | 339 (33%) |
|  | 1-2 per week | 608 (59%) |
|  | 3+ per week | 76 (7.4%) |
| Body mass index | Healthy | 537 (52%) |
|  | Obese | 135 (13%) |
|  | Overweight | 321 (31%) |
|  | Underweight | 30 (2.9%) |
| Sleep per night | Less than 5 hrs | 65 (6.4%) |
|  | 6-8 hours | 847 (83%) |
|  | 8+ hours | 111 (11%) |
| Smoking status | Never | 778 (76%) |
|  | Occasionally in the past | 79 (7.7%) |
|  | Frequently in the past | 76 (7.4%) |
|  | Regularly | 90 (8.8%) |
| Alcohol consumption | Never | 66 (6.5%) |
|  | In the past | 68 (6.6%) |
|  | Monthly or less | 151 (15%) |
|  | 2-4 per month | 281 (27%) |
|  | 2-3 per week | 253 (25%) |
|  | 4+ per week | 204 (20%) |
| Caffeine consumption | None | 173 (17%) |
|  | 1-2 cups per day | 524 (51%) |
|  | 3 cups or more per day | 326 (32%) |
| Leisure time | 0-1 hours per week | 184 (18%) |
|  | 1-4 hours per week | 371 (36%) |
|  | 5-9 hours per week | 294 (29%) |
|  | 10-14 hours per week | 94 (9.2%) |
|  | 15+ hours per week | 80 (7.8%) |
| Reading time | 1 hour or less a week | 170 (17%) |
|  | 2-4 hours per week | 438 (43%) |
|  | 5-9 hours per week | 270 (26%) |
|  | 10+ hours per week | 145 (14%) |
| Mental challenges | None | 379 (37%) |
|  | 0-1 hours per week | 344 (34%) |
|  | 2-4 hours per week | 239 (23%) |
|  | 5+ hours per week | 61 (6.0%) |
| Time with friends | None | 74 (7.2%) |
|  | 1-2 times per week | 634 (62%) |
|  | 3-4 times per week | 224 (22%) |
|  | 5+ times per week | 91 (8.9%) |
| Time with family | None | 233 (23%) |
|  | 1-2 times per week | 540 (53%) |
|  | 3-4 times per week | 119 (12%) |
|  | 5+ times per week | 131 (13%) |
| Volunteering | None | 697 (68%) |
|  | 1-2 times per week | 291 (28%) |
|  | 3+ time per week | 35 (3.4%) |
| Marital Status | Single | 301 (29%) |
|  | Divorced | 49 (4.8%) |
|  | De-facto | 110 (11%) |
|  | Married | 536 (52%) |
|  | Separated | 21 (2.1%) |
|  | Widow(er) | 6 (0.6%) |

*Notes.* N = 1023

**Table S3. Reliabilities for multi-item measures.**

| **Measure** | **Cronbach's Alpha** | **95% CI** | |
| --- | --- | --- | --- |
|  |  | **LL** | **UL** |
| COMPAS-W | 0.893 | 0.882 | 0.901 |
| NEO-FFI Extraversion | 0.806 | 0.789 | 0.821 |
| NEO-FFI Neuroticism | 0.879 | 0.868 | 0.889 |
| NEO-FFI Conscientiousness | 0.845 | 0.831 | 0.858 |
| NEO-FFI Openness | 0.752 | 0.73 | 0.771 |
| NEO-FFI Agreeableness | 0.755 | 0.732 | 0.774 |
| ERQ Suppression | 0.442 | 0.385 | 0.494 |
| ERQ Reappraisal | 0.724 | 0.692 | 0.748 |

**Table S4: Final cross-sectional model results**

| Coefficients: |  |  |  |  |  |
| --- | --- | --- | --- | --- | --- |
|  | **Estimate** | **Std. Error** | **t value** | **Pr(>\|t\|)** |  |
| **(Intercept)** | **62.94** | **2.71** | **23.24** | **< 0.001** | ******* |
| **Age** | **0.05** | **0.02** | **2.62** | **0.009** | ****** |
| Sex [Male] | 0.39 | 0.38 | 1.02 | 0.307 |  |
| Zygosity [MZ] | 0.51 | 0.34 | 1.50 | 0.133 |  |
| Education [Trade] | 0.43 | 0.71 | 0.61 | 0.542 |  |
| Education [Diploma/Certificate] | 0.30 | 0.49 | 0.61 | 0.543 |  |
| Education [Graduate] | 0.41 | 0.47 | 0.88 | 0.381 |  |
| **Education [Postgraduate]** | **1.12** | **0.53** | **2.11** | **0.035** | ***** |
| Education [Other] | 1.68 | 2.59 | 0.65 | 0.518 |  |
| Marital Status [Divorced] | -1.03 | 0.86 | -1.19 | 0.236 |  |
| Marital Status [De-facto] | -0.23 | 0.61 | -0.39 | 0.698 |  |
| Marital Status [Married] | 0.54 | 0.48 | 1.12 | 0.262 |  |
| Marital Status [Separated] | -0.78 | 1.21 | -0.64 | 0.519 |  |
| Marital Status [Widowed] | -1.83 | 2.16 | -0.85 | 0.396 |  |
| BMI [Obese] | -0.92 | 0.52 | -1.77 | 0.078 | . |
| BMI [Overweight] | -0.18 | 0.38 | -0.49 | 0.626 |  |
| BMI [Underweight] | 0.48 | 0.96 | 0.50 | 0.614 |  |
| **Neuroticism** | **-0.59** | **0.03** | **-22.29** | **< 0.001** | ******* |
| **Extraversion** | **0.45** | **0.03** | **13.85** | **< 0.001** | ******* |
| **Conscientiousness** | **0.52** | **0.03** | **15.86** | **< 0.001** | ******* |
| **Openness** | **0.13** | **0.03** | **4.43** | **< 0.001** | ******* |
| Agreeableness | 0.02 | 0.03 | 0.60 | 0.548 |  |
| **Cognitive Reappraisal** | **1.64** | **0.19** | **8.64** | **< 0.001** | ******* |
| Emotional Suppression | -0.25 | 0.15 | -1.69 | 0.090 | . |
| Absenteeism | 0.01 | 0.004 | 0.13 | 0.897 |  |
| Presenteeism | -0.01 | 0.01 | 1.25 | 0.212 |  |
| Motor Coordination | -0.27 | 0.27 | -1.02 | 0.309 |  |
| Inhibition | 0.11 | 0.28 | 0.399 | 0.690 |  |
| Sustained Attention | -0.12 | 0.32 | -0.37 | 0.713 |  |
| **Cognitive Flexibility** | **0.71** | **0.33** | **2.14** | **0.032** | ***** |
| **Working Memory** | **0.49** | **0.17** | **2.92** | **0.004** | ****** |
| Happy Reaction Time | -0.01 | < 0.01 | -0.43 | 0.666 |  |
| Time with Family [1-2/wk] | -0.24 | 0.41 | -0.58 | 0.559 |  |
| Time with Family [3-4/wk] | -0.41 | 0.59 | -0.70 | 0.483 |  |
| Time with Family [5+/wk] | -0.65 | 0.58 | -1.12 | 0.262 |  |
| Time with Friends [1-2/wk] | -0.34 | 0.64 | -0.54 | 0.591 |  |
| Time with Friends [3-4/wk] | -0.29 | 0.73 | -0.40 | 0.692 |  |
| Time with Friends [5+/wk] | 0.61 | 0.88 | 0.69 | 0.487 |  |
| Smoking Status [Occasionally Past] | 0.02 | 0.61 | 0.03 | 0.976 |  |
| Smoking Status [Frequently Past] | 0.13 | 0.63 | 0.21 | 0.831 |  |
| **Smoking Status [Current]** | **-1.43** | **0.59** | **-2.40** | **0.017** | ***** |
| Leisure Time [1-4hr/wk] | 0.23 | 0.48 | 0.49 | 0.621 |  |
| Leisure Time [5-9hr/wk] | 0.69 | 0.51 | 1.35 | 0.176 |  |
| Leisure Time [10-14hr/wk] | 0.83 | 0.68 | 1.21 | 0.226 |  |
| **Leisure Time [15+hr/wk]** | **1.89** | **0.74** | **2.54** | **0.011** | ***** |
| Exercise Frequency [1-2/wk] | 0.05 | 0.49 | 0.09 | 0.925 |  |
| Exercise Frequency [3-4/wk] | 0.30 | 0.51 | 0.58 | 0.558 |  |
| Exercise Frequency [5+/wk] | -0.26 | 0.56 | -0.46 | 0.644 |  |
| Sleep Hours [6-8/night] | -0.23 | 0.68 | -0.343 | 0.731 |  |
| Sleep Hours [8+/night] | -0.72 | 0.83 | -0.87 | 0.385 |  |
| Fruit & Vegetable intake [5-6/wk] | 0.66 | 0.47 | 1.40 | 0.163 |  |
| Fruit & Vegetable intake [7+/wk] | 0.41 | 0.47 | 0.87 | 0.382 |  |
| Volunteering [1-2/wk] | 0.44 | 0.37 | 1.17 | 0.241 |  |
| Volunteering [3+/wk] | -0.99 | 0.90 | -1.11 | 0.269 |  |

*Notes.* N = 1017

**Table S5. Final repeated measures model results**

| Coefficients: |  |  |  | |  |  |
| --- | --- | --- | --- | --- | --- | --- |
|  | Estimate | Std. Error | | t value | Pr(>\|t\|) |  |
| (Intercept) | 18.57 | 4.22 | 4.40 | | 0.000 | *** |
| **Baseline Wellbeing** | **0.61** | **0.04** | **15.09** | | **< 2e-16** | *** |
| Age | 0.04 | 0.02 | 1.81 | | 0.070 | . |
| Sex [Male] | 0.75 | 0.48 | 1.56 | | 0.119 |  |
| Zygosity [MZ] | -0.54 | 0.43 | -1.27 | | 0.203 |  |
| Education [Trade] | -0.05 | 0.89 | -0.06 | | 0.951 |  |
| Education [Diploma/Certificate] | 0.14 | 0.62 | 0.23 | | 0.815 |  |
| Education [Graduate] | 0.64 | 0.59 | 1.09 | | 0.276 |  |
| **Education [Postgraduate]** | -0.03 | 0.67 | -0.04 | | 0.968 |  |
| Education [Other] | 1.66 | 3.24 | 0.51 | | 0.607 |  |
| Marital Status [Divorced] | 0.86 | 1.08 | 0.80 | | 0.427 |  |
| Marital Status [De-facto] | 1.15 | 0.76 | 1.51 | | 0.131 |  |
| Marital Status [Married] | 0.33 | 0.61 | 0.55 | | 0.584 |  |
| Marital Status [Separated] | 1.58 | 1.50 | 1.05 | | 0.294 |  |
| Marital Status [Widowed] | 4.07 | 2.70 | 1.51 | | 0.131 |  |
| BMI [Obese] | -0.99 | 0.65 | -1.52 | | 0.130 |  |
| BMI [Overweight] | 0.25 | 0.47 | 0.52 | | 0.602 |  |
| BMI [Underweight] | 0.58 | 1.20 | 0.49 | | 0.627 |  |
| **Neuroticism** | -0.08 | 0.04 | -1.92 | | 0.055 | . |
| **Extraversion** | **0.20** | **0.04** | **4.54** | | **0.000** | *** |
| **Conscientiousness** | **0.13** | **0.05** | **2.85** | | **0.004** | ** |
| **Openness** | 0.07 | 0.04 | 1.83 | | 0.068 | . |
| Agreeableness | 0.00 | 0.04 | 0.02 | | 0.981 |  |
| **Cognitive Reappraisal** | 0.37 | 0.25 | 1.50 | | 0.133 |  |
| Emotional Suppression | -0.04 | 0.19 | -0.20 | | 0.846 |  |
| Absenteeism | 0.00 | 0.00 | -0.05 | | 0.959 |  |
| Presenteeism | **-0.03** | **0.01** | **2.33** | | **0.020** | * |
| Motor Coordination | 0.23 | 0.34 | 0.68 | | 0.499 |  |
| Inhibition | -0.18 | 0.35 | -0.51 | | 0.612 |  |
| Sustained Attention | 0.67 | 0.40 | 1.68 | | 0.093 | . |
| **Cognitive Flexibility** | 0.75 | 0.41 | 1.83 | | 0.068 | . |
| **Working Memory** | -0.26 | 0.21 | -1.24 | | 0.217 |  |
| Happy Reaction Time | 0.00 | 0.00 | 0.12 | | 0.902 |  |
| Time with Family [1-2/wk] | -0.52 | 0.51 | -1.02 | | 0.307 |  |
| Time with Family [3-4/wk] | -0.03 | 0.73 | -0.04 | | 0.971 |  |
| Time with Family [5+/wk] | -0.80 | 0.73 | -1.10 | | 0.271 |  |
| Time with Friends [1-2/wk] | 1.53 | 0.80 | 1.91 | | 0.056 | . |
| Time with Friends [3-4/wk] | **2.04** | **0.92** | **2.23** | | **0.026** | * |
| Time with Friends [5+/wk] | 1.17 | 1.11 | 1.06 | | 0.290 |  |
| Smoking Status [Occasionally Past] | 0.15 | 0.76 | 0.20 | | 0.840 |  |
| Smoking Status [Frequently Past] | 0.70 | 0.79 | 0.89 | | 0.374 |  |
| **Smoking Status [Current]** | 0.88 | 0.74 | 1.18 | | 0.238 |  |
| Leisure Time [1-4hr/wk] | -0.47 | 0.60 | -0.79 | | 0.430 |  |
| Leisure Time [5-9hr/wk] | 0.03 | 0.64 | 0.05 | | 0.962 |  |
| Leisure Time [10-14/wk] | 0.09 | 0.85 | 0.10 | | 0.917 |  |
| **Leisure Time [15+hr/wk]** | -2.04 | 0.93 | -2.19 | | 0.028 | * |
| Exercise Frequency [1-2/wk] | 0.81 | 0.61 | 1.33 | | 0.185 |  |
| Exercise Frequency [3-4/wk] | -0.53 | 0.63 | -0.84 | | 0.403 |  |
| Exercise Frequency [5+/wk] | 0.90 | 0.70 | 1.29 | | 0.198 |  |
| Sleep Hours [6-8hr/night] | 0.14 | 0.85 | 0.16 | | 0.873 |  |
| Sleep Hours [8+/night] | 1.74 | 1.03 | 1.68 | | 0.094 | . |
| Fruit & Vegetable intake [5-6/wk] | **1.58** | **0.59** | **2.66** | | **0.008** | ****** |
| Fruit & Vegetable intake [7+/wk] | **1.69** | **0.59** | **2.87** | | **0.004** | ****** |
| Volunteering [1-2/wk] | -0.51 | 0.47 | -1.10 | | 0.273 |  |
| Volunteering [3+/wk] | -1.01 | 1.12 | -0.90 | | 0.371 |  |
| **DLE Relationships** | **0.19** | **0.08** | **2.29** | | **0.022** | ***** |
| DLE Family | -0.04 | 0.10 | -0.35 | | 0.723 |  |
| **DLE Work** | **0.17** | **0.05** | **3.49** | | **0.001** | *** |
| DLE Lifestyle | 0.10 | 0.06 | 1.66 | | 0.097 | . |
| **DLE Trauma** | **0.55** | **0.16** | **3.48** | | **0.001** | *** |

*Notes.* N = 1017.
